# Retrospective study on the incidence of *pseudoexfoliatio lentis* and related complications in a cohort of patients from the Island of Ischia: medico-legal and ergophthalmology considerations

**DOI:** 10.22336/rjo.2021.46

**Published:** 2021

**Authors:** Mauro Salducci, Francesco Maiorano, Carmen Rachele Maione, Concetta Nappi, Marzio Di Meglio

**Affiliations:** * Faculty of Medicine and Dentistry, Department of Sense Organs, Medical Clinical in Forensic Ophthalmology, Sapienza University Rome, Italy; Umberto I Hospital, Italy; ** Department of Radiological, Oncological and Anatomo-Pathological Sciences, Sapienza University of Rome, Italy; *** Di Meglio Eye Center, Forio D’Ischia (NA), Italy; **** Department of Ophthalmology, Maria Rosaria Nursing Home, Pompeii (NA), Italy

**Keywords:** *pseudoexfoliatio lentis* or pseudoexfoliation syndrome (PEX), glaucoma, capsule-IOL (Intra Ocular lens) complex dislocation and subluxation in vitreous chamber (L-SL in CV), capsule-IOL complex resuspension, (PPV) pars plana vitrectomy, surgically induced astigmatism (SIA)

## Abstract

The population of the island has lived for centuries almost isolated from the rest of the continental population. In the seventh century BC, it was the first Greek colony in the southern Italy and was colonized by the Eubei, then it was the turn of the Roman rule. In the Middle Ages, the island was the victim of many raids of the Barbary pirates. Only in the last decades of the 800, did the island begin to open to tourism. However, it had a strong setback with the terrible earthquake of Casamicciola, in 1883. Tourism had gradually resumed in the second post-war period. These episodes have contributed to bringing people extraneous to the autochthonous community to the island. As in most of the coastal populations of the Mediterranean basin, there is a very high incidence of pseudoexfoliation lentis in the population of Ischia, which is a syndrome that often complicates in a challenging way the surgical intervention for the removal of cataracts.

## Introduction

In collaboration with Prof. Salducci, specialist and expert in Medical and Labor Ophthalmology, and with the local ophthalmology group located in Forio of Ischia (NA) and directed by Dr. Di Meglio, we performed a retrospective study on 3802 cataract operations performed on patients from the island of Ischia (NA) in the last 10 years and on complications related to *pseudoexfoliatio lentis* known as pseudoexfoliation syndrome (PEX), also in relation to the occupational and visual rehabilitation of the patients (**Table 1**). It should be noted that PEX presents several difficulties in the management of both pre, intra, and post-operative patient, as well as several medical and legal implications. Only a very experienced surgeon can manage these patients but must also be able to explain the complications and comorbidities to the patient and must customize the informed consent form for surgery in order not to be susceptible to claims for compensation. Most of the cataract cases that came to our observation and were operated on, were nuclear or corticonuclear and many presented as clinically evolved and severe, brownish and/ or total white. Patients not originating from the island of Ischia were excluded from the study [**1**-**5**]

**Tabel 1:** Case study

Year	Cataracts	PEX monol.	PEX bilat.	Glaucoma	Dislocation	Donesi	Second surgery	IOL exchange
2011	390	55	44	85	6	25	6	3
2012	425	62	57	73	8	29	10	1
2013	496	51	53	63	11	31	11	0
2014	427	56	61	81	8	29	9	0
2015	385	46	53	68	12	33	13	0
2016	415	51	56	72	7	40	7	0
2017	378	54	55	61	10	27	10	0
2018	386	59	61	67	13	28	13	0
2019	415	56	62	61	5	19	6	0
2020	85	15	29	38	1	9	1	0

**Fig. 1:**
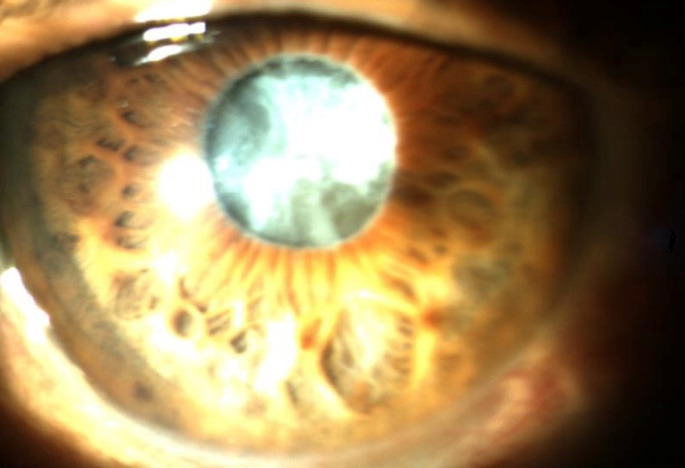
White cataract, pupillary edge fibrosis, PEX

In addition, a simultaneous diagnosis of surgically relevant cataract in a very advanced stage (**Fig. 1**) and glaucoma (17.57%), often in a terminal stage, was frequently made.

**Fig. 2:**
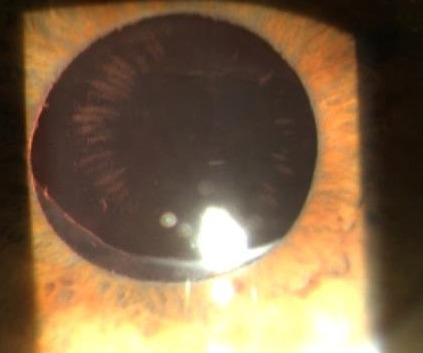
Rare image of deposits of pseudo-exfoliative material on the optical plate of an IOL

In many cases, we detected both dislocation and glaucoma, as well as deposits of pseudo-exfoliative material, also on the IOL (**Fig. 2**).

## Materials and methods

**Fig. 3:**
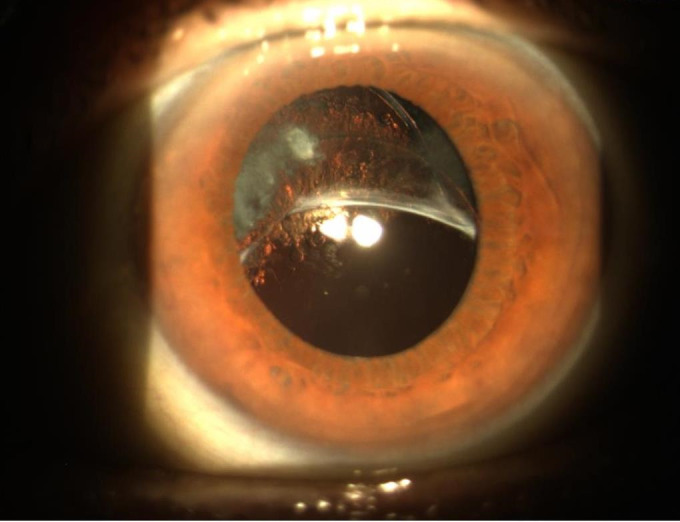
Lower subluxation of the capsular bag containing the IOL due to the loss of the upper zoning fibers

**Fig. 4:**
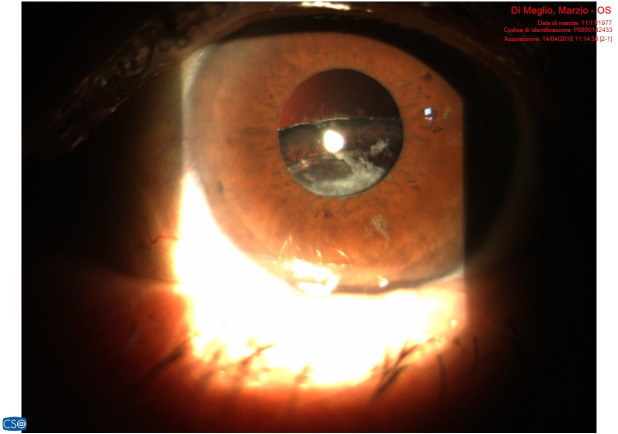
Lower dislocation of the capsular bag containing the IOL

We noted that the percentage of dislocations/ subluxations (L/ SL) did not differ greatly between patients operated in early-stage phacoemulsification compared to those operated in late stage. We attributed this homogeneity to several factors, first, the subluxations (**Fig. 3**) and the dislocations (**Fig. 4**), which were due to the progressive failure of the zonular fibers caused by the PEX, and secondly, to the fact that the most expert surgeons were then well acquainted with the pathology and possible complications, so that in most complex cases they performed a wide capsulorhexis and dislocated the nucleus outside the sac, and then proceed to phacoemulsification on the iris plane. In order to avoid damaging the endothelium we obviously used generous doses of viscoelastic adhesive type in our surgeries [**9**,**10**].

All patients preoperatively underwent a complete eye examination, anterior segment with the CSO 990 Digital system, endothelial cell counts with the CSO SP02 endothelial microscope, and optical biometry, or ultrasonic biometry with the Nidek AL-Scan biometer (both for surgeries in which IOL exchange was planned and in cases in which resuspension was planned) [**6**-**8**].

## Results

We noted that compared to the use of a lens with iris enclavation, not using corneal sutures, we generated a very low surgically induced astigmatism (SIA), the patient had very little ocular discomfort postoperatively and returned to normal life activities in about 5 days. In most cases, the natural sight postoperatively was equal to or greater than 6/ 10, allowing driving without the obligation of wearing the lens. It is true that they were all retired, but many of them were devoted to agriculture and returned in less than a week to their heavy life habits without complications. We found a substantial stationarity of visual performance even in the controls, carried out every six months [**11**-**13**] (**Table 2**).

**Tabel 2:** The natural and correct visual acuity in case of subluxation and dislocation of the capsule-IOL complex both in the pre- and post-operative period

Total cases	Total dislocation with vvp.	Resuspension	Total interventions
81	16	70	86
	n. pre-op v. a. counting fingers to one meter	n. pre-s v. a. 2/ 10	
	b. c. a. pre-s 6/ 10 sf + 13	b. c. v. a. pre-s. 3/ 10	
	n. a. v. p. s. 6/ 10	n. a. v. p. s. 6/ 10	
	b. c. a. p. s. 8/ 10	b. c. a. p. s. 7-8/ 10	
Average age 75 years (63-90)			

It should be noted that in the OP, 3 patients presented loss of visual function for other causes: 1 senile exudative maculopathy - 3 years OP; 1 ischemic event at the expense of the n. o. - 7 years OP; 1 occlusion vecr. - 5 years OP.

## Discussion

**Fig. 5:**
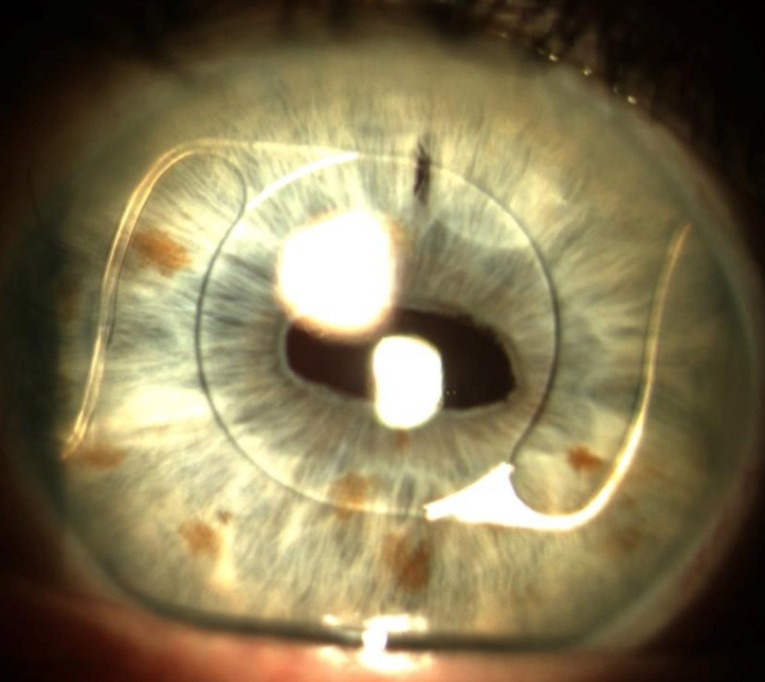
Anterior chamber IOL implanted after complicated cataract surgery

In the early days, especially for complete dislocations of the capsule/ IOL complex, we provided ppv. 23G, associated with IOL-exchange, implanting iris-enclaving IOLs. We have never used angular fixation IOLs (**Fig. 5**) as in the case of a patient who emigrated to the USA and was operated on in New York 15 years before, and who came to our attention for an L in the adelphic eye [**14**,**15**].

Despite the good results, in recent years we have moved to a more conservative approach: in case of subluxation in fact we identified the quadrant where the zonula has failed, we performed a minimal peritomy and a scleral flap with a limbus-based flap at 50% of the thickness and a paracentesis at 180° from the scleral flap. Afterwards, we filled the anterior chamber with cohesive viscoelastic material, passed the prolene straight needle inferiorly to the complex, passed it through and moved to the anterior chamber. Through the paracentesis, we retrieved the straight needle with the help of a 30G hollow needle, extracted the prolene straight needle from the anterior chamber, turned it 180° and reinserted it into the c. a., passing this time above the capsule-anus complex of the IOL, we extracted it from the sclera, always with the help of the 30G guide needle. We proceeded to tie the two ends of the prolene thread previously passed at 2.5 and 2.8 mm from the limbus, carefully adjusting the tension in order to center the optical dish on the pupillary foramen. Then, we put back in place the scleral flap, in order to protect and cover the nodes and proceed to close the conjunctiva with one or two detached stitches in Vicril 8.0. Then, we removed the viscomaterial through paracentesis and hydrosealed it. If over the years, the zonula should further collapse, or in the rare cases in which the IOL centering was not satisfactory in the postoperative period, we proceeded to perform the same procedure at 180° from the first.

In recent years, even in the case of total dislocation of the capsule/ IOL complex in CV, we proceeded with a 23G or 25G PPV and resuspension of the complex to the sclera. In our case histories, we did not find significant complications. In fact, the postoperative course was regular, we noticed that compared to an IOL-exchange the loss of endothelial cells and the surgically induced astigmatism (both) were minimal [**16**-**18**].

**Medico legal considerations**

From the point of view of medico-legal principles in ophthalmology, we obviously believe that the closed-bulb procedure is much safer for the patient and the physician, since the former is not exposed to the risk of expulsive hemorrhage or excessive SIA. In the surgery of cataracts complicated by PEX and in the surgery and its complications, Article 2236 of the Civil Code that expressly states “If the service involves the solution of technical problems of special difficulty, the provider is not liable for damages, except in cases of fraud or gross negligence”, is in fact fully applied. For greater protection, we added the following wording to the informed consent: “eye affected by PEX, therefore at high risk of L/ SL of the crystalline lens in the vitreous chamber during the intervention”. We warned that even after a perfectly successful intervention, there is a high risk of L/ SL of the capsular sac + IOL complex with the need to undergo multiple surgeries [**19**-**21**].

## Conclusions

Therefore, as stated above, the surgical procedures in the case of PEX can nowadays be considered reliable from various points of view. The clinical as well as the surgical methods implemented were highly reliable, but also the medico-legal and rehabilitation, as all the patients considered in this study have been functionally rehabilitated from a visual point of view, so that they could easily return to their previous occupations and therefore with lower costs for the public social security funds of the Italian state, in terms of a lower number of visually impaired retirees.

**Conflict of Interest statement**

The authors state no conflict of interest.

**Informed Consent and Human and Animal Rights statement**

Informed consent has been obtained from all individuals included in this study.

**Authorization for the use of human subjects**

Ethical approval: The research related to human use complies with all the relevant national regulations, institutional policies, is in accordance with the tenets of the Helsinki Declaration, and has been approved by the review board of Sapienza University of Rome, Italy.

**Acknowledgements**

None.

**Sources of Funding**

None.

**Disclosures**

None.
